# Matrix Metalloproteinase-2 and -9 Expression in the Epiligament of the Medial Collateral and Anterior Cruciate Ligament in Human Knees: A Comparative Study

**DOI:** 10.7759/cureus.3550

**Published:** 2018-11-05

**Authors:** Georgi P Georgiev, Boycho Landzhov, Georgi Kotov, Svetoslav A Slavchev, Alexandar Iliev

**Affiliations:** 1 Orthopaedics and Traumatology, Queen Giovanna Hospital, Sofia, BGR; 2 Anatomy, Histology and Embryology, Medical University of Sofia, Sofia, BGR; 3 Orthopaedics and Traumatology, Medical University of Sofia, Sofia, BGR

**Keywords:** epiligament, medial collateral ligament, anterior cruciate ligament, matrix metalloproteinase-2 and 9, human knee

## Abstract

Aim

Ninety percent of knee ligament injuries involve the medial collateral ligament (MCL) and the anterior cruciate ligament (ACL) of the knee joint. Matrix metalloproteinases (MMPs) are a large group of calcium- and zinc-dependent endopeptidases responsible for cleaving and rebuilding various connective tissue components. Previous studies showed that MMP-2 and 9 have a significant effect on the healing process of injured ligaments. Therefore, the aim of this study was to evaluate for the first time in literature the expression and localization of MMP-2 and 9 in the epiligament (EL) and the ligament tissue of the MCL and the ACL of the human knee joint in order to assess their role in ligament healing.

Materials and methods

For the present study, we used histological material from the mid-portion of the MCL and the ACL of 14 knee joints from fresh cadavers. For the purpose of the immunohistochemical analysis, we used primary polyclonal antibodies against MMP-2 and 9. The obtained results were evaluated semi-quantitatively through ImageJ.

Results

Immunoreactivity for MMP-2 was predominantly positive (2+) in the EL of the MCL and remained mostly negative (0) in the ligament tissue. The expression of MMP-9 was mostly low-positive (1+) in the EL of the MCL and almost entirely negative (0) in the ligament tissue. In the EL of the ACL, the immunohistochemical expression of MMP-2 was predominantly low-positive (1+) and that of the MMP-9 was read as mostly low-positive (1+). Expression of the two enzymes in the ligament tissue was similar to the MCL.

Conclusion

The present study is the first comparison of the expression of the aforementioned MMPs in the EL tissue of the MCL and the ACL in human knees, which may play a key role in physiological and pathophysiological processes such as tissue healing and repair and basement membrane degradation.

## Introduction

The medial collateral ligament (MCL) of the knee joint is one of the most commonly injured ligaments of the knee [1-2]. Ninety percent of knee ligament injuries involve the MCL or the anterior cruciate ligament (ACL) [2-3]. The incidence of injuries of these ligaments has increased in recent years and presents a commonly encountered problem in modern sports medicine [3-5]. Moreover, the ACL has a poor healing ability, especially in comparison with the MCL, which can heal relatively well [6-7].

The thin connective tissue layer enveloping the ligament was first described as the ‘epiligament’ (EL) by Bray et al. [8]. In 2017, Georgiev et al. [4] hypothesised that the EL of the MCL has specific characteristics and suggested that the fibroblasts in particular, together with the abundant blood vessels are essential for its nutrition and healing. These data were in line with the previous studies on the MCL in rat model [9-12, 13-17]. According to Georgiev and Vidinov [13-15] and Georgiev et al. [9-12] the EL of the MCL is a donor of fibroblasts, progenitor cells, and blood vessels during the process of ligament recovery. In contrast, the biological basis for failure of the human ACL to heal after rupture is still unknown.

Matrix metalloproteinases (MMPs), also known as matrixins, are a large group of calcium- and zinc-dependent endopeptidases responsible for cleaving and rebuilding connective tissue components such as collagen, elastin, gelatin, and casein [18-21]. These enzymes are produced by various connective tissue cells, such as fibroblasts, endothelial cells, osteoblasts, macrophages and others [16]. According to literature data, MMP-2 and 9 are involved in complex processes, such as tissue remodeling and repair, basement membrane degradation, healing of acute MCL and ACL tears, tumour invasion, angiogenesis, etc. [4, 22-23]. Taking into consideration the difference in the healing potential between the MCL and the ACL, we were interested to evaluate the expression of MMPs in the EL of the two ligaments, in order to determine whether there were any variations.

Therefore, the aim of this study was to evaluate for the first time in literature the expression and localization of MMP-2 and 9 in the EL and the ligament tissue of the MCL and ACL in the human knee joint in order to assess their role in ligament healing.

## Materials and methods

Tissue preparation

For the present study, we used 14 knee joints from fresh cadavers from the autopsy material available at the Department of Anatomy, Histology and Embryology at the Medical University of Sofia. Eight specimens were female and six were male with a mean age at death of 64.5 years old. No medical or surgical history for trauma of the knees of the cadavers was available. The study was approved by the Medical Legal Office, the Local Ethics Committee, and the Institutional Review Board (No. 4866).

After skin incision, the underlying subcutaneous tissue was dissected to expose the MCL of the knee joint. The MCL and the external surface of the surrounding EL were precisely dissected, the points of insertion of the ligament were excised, and the mid portion of the MCL was immediately fixed in 10% formalin solution (Merck Catalog No. 1040031000, Merck KGgA, Darmstadt, Germany) for 24 hours and were then dehydrated in increasing concentrations of ethanol (70%, 80%, 95%, 100%) (Merck Catalog No. 1009835000). After opening the capsule of the knee joint, the mid portion of the ACL was processed and fixed in the previously described way. Next, ethanol was removed using cedar oil until the samples became translucent. The samples were then rinsed in xylene (Merck Catalog No. 1082984000) and embedded in paraffin (Merck Catalog No. 1071511000).

Immunohistochemistry protocol

Each paraffin block (*n* = 28, 14 of each ligament) was cut on a microtome (Leica, Wetzlar, Germany) into 5 µm thick sections which were mounted on slides previously coated with chrome-gelatin. Next, we randomly selected 10 slides per paraffin block, thus obtaining a total number of 140 slides of each ligament. Sections were deparaffinized, rehydrated with ethanol (100%, 95%, 80%, 70%) (Merck Catalog No. 1009835000), and washed in 0.1 M phosphate buffer (Merck Catalog No. 1465920006), pH 7.4, at room temperature. Endogenous peroxidase activity was blocked with 3% hydrogen peroxide (H_2_O_2_) for 10 minutes at room temperature. The sections were rinsed in phosphate-buffered saline (PBS) (Merck Catalog No. 6505-4L) and nonspecific binding sites were blocked with Super Block (ScyTek Catalog No. AAA125, ScyTek Laboratories, Inc., Logan, Utah, USA) for five minutes. Primary rabbit anti-human polyclonal antibodies against MMP-2 (Sigma Aldrich Catalog No. HPA001939, Sigma Aldrich Chemie GmbH, Taufkirchen, Germany) and MMP-9 (Sigma Aldrich Catalog No. ABT544) at a dilution 1:500 were added and the sections were incubated overnight at 4ºC, rinsed in PBS (Merck Catalog No. 6505-4L), and incubated with biotinylated goat anti-rabbit immunoglobulin G (IgG) (UltraTek Anti-Rabbit, ScyTek Catalog No. UAR125) for 10 minutes at room temperature. Sections were rinsed as before and incubated with streptavidin-HRP (UltraTek HRP Anti-Rabbit, ScyTek Catalog No. UHR125) for 10 minutes at room temperature. Antibody binding was visualized using 3,3′-diaminobenzidine (DAB) (Sigma Aldrich Catalog No. D12384) as chromogen for 10 minutes. Sections were counterstained with hematoxylin (Merck Catalog No. 1051741000), dehydrated in increasing concentrations of ethanol (70%, 80%, 95%, 100%) (Merck Catalog No. 1009835000), cleared in xylene (Merck Catalog No. 1082984000), and cover-slipped with Canada balsam (Sigma Aldrich Catalog No. C1795). Sections used as controls were incubated in the way previously described, but omitting the primary or secondary antibody. All controls were negative. The immunohistochemical staining of all sections was conducted under the same conditions. Photomicrographs of representative fields of the immunohistochemical staining were obtained using an Olympus CX 21 microscope fitted with an Olympus C5050Z digital camera (Olympus Optical Co., Ltd., Tokyo, Japan).

Semi-quantitative analysis

For semi-quantitative analysis of the expression of MMP-2 and -9, we used the software ImageJ 1.52a. The intensity of staining was assessed through the IHC Profiler plugin, according to the well-established protocol. As indicated above, we used 140 slides per ligament and analyzed at least 10 randomly selected visual fields on each slide. The IHC Profiler assigned a score to each visual field in a four tier system—high positive (3+), positive (2+), low positive (1+), and negative (0). The immunohistochemical expression in the EL and the ligament tissue of the MCL and the ACL were presented as percentages of the respective scores as calculated by the IHC Profiler.

## Results

Immunohistochemical analysis of MMP-2 and -9 expression in the MCL

**Figure 1:**
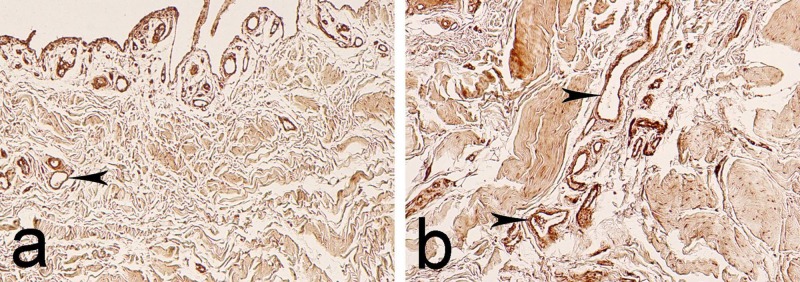
Immunohistochemical expression of matrix metalloproteinase-2 (MMP-2) in the epiligament (EL) of the medial collateral ligament (MCL). Arrowheads - immunoreactivity in the adventitia of blood vessels. a. Magnification: 200x b. Magnification: 400x

The immunohistochemical analysis of the expression of MMP-2 and 9 revealed a heterogeneous distribution of the enzymatic activity of the two enzymes in the EL and in the ligament tissue of the MCL. The positive staining for MMP-2 was distributed along the entire thickness and was best expressed in the adventitia of blood vessels in the EL, as well as in the perivascular zone (Figures 1a-1b).

**Figure 2:**
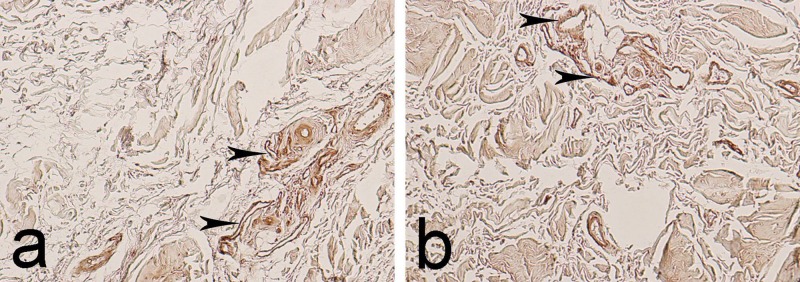
Immunohistochemical expression of matrix metalloproteinase-9 (MMP-9) in the epiligament (EL) of the medial collateral ligament (MCL). Arrowheads - immunoreactivity in the adventitia of blood vessels. a. Magnification: 400x b. Magnification: 400x

The semi-quantitative analysis revealed positive (2+) expression in 70% of visual fields and low-positive (1+) expression in the remaining 30%. Perhaps an interesting finding was that some fibroblasts in the ligament tissue also stained positive for MMP-2. Immunoreactivity was calculated as negative (0) in 76% of visual fields and as low positive (+) in the remaining 24%. The enzymatic activity of MMP-9 in the EL was read as low positive (+) in 63% of visual fields, positive (2+) in 28%, and negative (0) in 9% of visual fields. As in the case of MMP-2, staining was best expressed in the adventitia of blood vessels and in the perivascular zone (Figures 2a-2b).

In the ligament tissue, the expression of MMP-9 was calculated as negative (0) in 88% of visual fields and low positive (1+) in 12% of visual fields, where it was observed mostly in the fibroblasts and in the adventitia of the few blood vessels.

Immunohistochemical analysis of MMP-2 and -9 expression in the ACL

**Figure 3:**
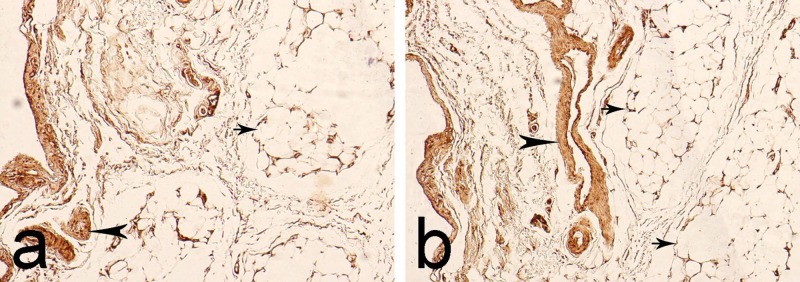
Immunohistochemical expression of matrix metalloproteinase-2 (MMP-2) in the epiligament (EL) of the anterior cruciate ligament (ACL). Arrowheads - immunoreactivity in the adventitia of blood vessels. Arrows - enzymatic activity in the fat tissue. a. Magnification: 400x b. Magnification: 400x

In the EL of the ACL, we observed MMP-2 expression in fibroblasts, fat tissue cells, and the adventitia of blood vessels (Figures 3a-3b).

**Figure 4:**
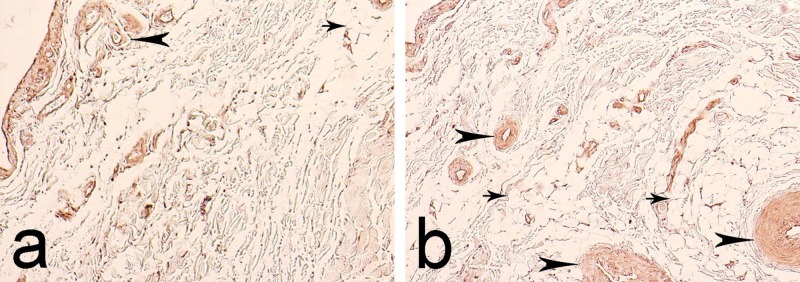
Immunohistochemical expression of matrix metalloproteinase-9 (MMP-9) in the epiligament (EL) of the anterior cruciate ligament (ACL). Arrowheads - immunoreactivity in the adventitia of blood vessels. Arrows - enzymatic activity in the fat tissue. a. Magnification: 400x b. Magnification: 400x

In 74% of visual fields, immunoreactivity was read as low positive (1+), while in the remaining 26%, it was calculated as positive (2+). In the ligament tissue, enzymatic activity was predominantly negative (92% of visual fields). In certain areas, it was calculated as low positive (1+) (8% of visual fields). This low-positive reaction was observed in some fibroblasts located close to the border between the EL and the ligament tissue. The distribution of the immunoreactivity of MMP-9 was similar to that of MMP-2. In the EL, it was reported in the adventitia of blood vessels and the perivascular space and in the adipose tissue cells (Figures 4a-4b).

The semi-quantitative analysis revealed a low-positive reaction (1+) in 83% of visual fields and negative reaction (0) in 17% of visual fields. In the ligament tissue, enzymatic activity was read as negative (0) in 94% of visual fields and low positive (1+) in the remaining 6% of visual fields. This enzymatic expression in the ligament tissue was only noted in individual fibroblasts located close to the border between the ligament tissue and the EL.

## Discussion

The MMPs are a family of calcium- and zinc-dependent endopeptidases which play a key role in the breakdown and removal of extracellular matrix molecules from the tissue, a process that takes place during developmental stages such as growth and morphogenesis [19, 24]. MMPs are also responsible for cleaving and rebuilding of collagen, elastin, gelatin, and casein and are thus involved in tissue-remodeling and wound healing [19, 24-25]. Apart from physiological processes, MMPs have also been implicated in pathological conditions and diseases such as Alzheimer’s disease and other disorders of the central nervous system, atherosclerosis, arthritis, liver cirrhosis, tumors and their metastases [21].

The MMP family members have been classified into different subgroups with close characteristics. The classification recognizes collagenases (MMP-1, 8, 13, 18), gelatinases (MMP-2 and 9), stromelysins (MMP-3, 10, 11), elastases (MMP-7, 12), and membrane type MMPs (MT-MMPs, MMP-14, 15, 16, 17), and a group of unnamed members [24]. Gelatinases include MMP-2 and MMP-9. MMP-2 (Gelatinase A) and MMP-9 (Gelatinase B) are known to cleave native collagen type I, IV, V, VII, X, XI and XIV, elastin, fibronectin, and osteonectin [18]. MMP-2 and 9 participate in tissue remodeling and repair, as well as basement membrane degradation and are thus involved in the healing of acute tears [18]. Gelatinases cleave denatured collagen and intact collagen type IV in basal membranes [18]. Zitka et al. [21] reported that MMP-2 binds to intact collagen to prevent autolytic inactivation. In addition, MMP-9 cleaves a number of other physiological substrates, such as claudin-1, occluding and ZO-1 [26].

After comparing the expression of different MMPs in the MCL and the ACL, Zhou et al. [27] concluded that numerous MMPs could be associated with the differences in healing potential. They reported that MMP-2 activity was higher in the injured ACL than in the MCL, which could present one potential reason of ACL healing failure. Tang et al. [7] described a 6.3-fold increase in MMP-2 expression in fibroblasts in injured ACL in comparison to injured MCL. MMP-9, on the other hand, was up-regulated in the injured MCL but to a much lesser degree than in the injured ACL. Ishiguro et al. [28] reported the presence of promatrix metalloproteinase-9 positive cells in the perivascular area in the ruptured ACL and promatrix metalloproteinase-2 positive cells between irregular collagen bundles in stumps of this ligament. According to these authors the positive reaction of these MMPs could not be determined whether it was due to rapid degradation or as a result of the degradative changes. Zhang et al. [29] reported a higher expression of MMP-2 in MCL fibroblasts than in ACL fibroblasts. Furthermore, they suggested that the differential expression of MMPs in the MCL and in the ACL may be involved in the differential healing potential of the two ligaments. Previous studies by Georgiev et al. [9] and Iliev et al. [16-17] observed the distribution and expression of MMP-2 and MMP-9 in normal rat tissue and also during the process of healing after acute injury. They showed that fibroblasts in the EL of the MCL normally generate low levels of MMP-2 and 9. These studies also noted that there was a more intensive reaction of MMP-2 in comparison to MMP-9. During ligament healing, after grade III injury, high levels of MMP-2 were expressed during all early ligament repairs [17]. In contrast, MMP-9 expression diminished and on the 30th day after injury, it was observed predominantly on the border between the EL and the MCL and in the perivascular zones in the EL [17].

In the present study, we compared the immunohistochemical expression of MMP-2 and 9 in the EL and ligament tissue of the MCL and the ACL. Our data supported the findings of Zhang et al. [29], who reported stronger expression of MMP-2 in the MCL than in the ACL. On the other hand, Tang et al. [7] described a much stronger expression of MMP-2 in the ACL as opposed to the MCL after injury. The discrepancy in observations could stem from the fact that these authors studied the expression and distribution of the enzyme after traumatic damage to the ligament. The differential expression of MMP-9 between the two ligaments has not been specifically studied in the literature; most often, authors have chosen to assess a large number of other enzymes of the MMP family [7, 19, 21]. Herein, we reported that the enzymatic activity of MMP-9 was stronger in the MCL than in the ACL and was observed predominantly in the EL tissue.

Limitations of the current study existed and should be outlined. The visual quantification of immunohistochemical images is associated with significant inter- and intra-observer variation. We attempted to resolve this issue by using the IHC Profiler plugin for ImageJ software, which eliminates inter-observer visual perception bias. In fact, 88.6% of scoring determined by the IHC Profiler coincides with blinded manual scoring determined by trained pathologists (P < 0.0001, confidence interval, CI = 95%) [30].

## Conclusions

This study represents the first report on the immunohistochemical localization and distribution of the enzymes MMP-2 and 9 in the EL of the MCL and the ACL in humans. Furthermore, we demonstrated and compared the enzymatic activity of MMP-2 and 9. These findings confirm our previous observations in rat model and are thus a further piece of evidence of the important role of the enzymes of the MMP group for the normal function of the MCL and the ACL and their role for the difference in the healing potential between the two ligaments.
